# Asymmetric Functional Divergence of *alx4a* and *alx4b* in Iridophore Differentiation and Cranial Development in Nile Tilapia

**DOI:** 10.3390/cells15171512

**Published:** 2026-08-22

**Authors:** Hongsheng Shi, Fugui Fang, Jiawen Yao, Siyu Ju, Minghui Li, Deshou Wang

**Affiliations:** Integrative Science Center of Germplasm Creation in Western China (Chongqing) Science City, Key Laboratory of Freshwater Fish Reproduction and Development (Ministry of Education), Key Laboratory of Aquatic Science of Chongqing, School of Life Sciences, Southwest University, Chongqing 400715, China; qq3381818533@email.swu.edu.cn (H.S.); ffg2022@email.swu.edu.cn (F.F.); g093615@email.swu.edu.cn (J.Y.); 17362494986@163.com (S.J.)

**Keywords:** *alx4*, iridophores, skull, functional divergence

## Abstract

**Highlights:**

- The first teleost *alx4a*/*alx4b* double mutant model reveals unequal functional contributions of the two paralogs after genome duplication.
- *alx4a* is the predominant contributor to iridophore-associated structural coloration, while *alx4b* provides a weaker, context-dependent contribution.
- The two paralogs contribute unequally to cranial development, with double mutants showing reduced opercular calcein labeling.

**Simple Summary:**

Neural crest cells give rise to both the craniofacial skeleton and several pigment cell types. Nile tilapia possess two paralogs of the developmental regulator Alx4, but their relative functional contributions remain poorly understood. We generated fish lacking either copy alone or both copies together. Loss of *alx4a* reduced reflective structural coloration in specific body regions and altered cranial morphology, whereas loss of *alx4b* caused no obvious phenotype under the conditions examined. Fish lacking both genes showed widespread loss of reflective coloration and severe cranial abnormalities. No significant genotype-dependent differences were detected in the measured abundance of other chromatophore lineages, and no obvious difference in gross dorsal-fin spine formation was observed under the conditions examined. Gene expression analyses were consistent with a role for *alx4* in iridophore differentiation and maturation, although possible effects on iridophore survival cannot be excluded. These findings indicate that these two paralogous genes make unequal functional contributions, with *alx4a* playing the predominant role and *alx4b* retaining a weaker contribution, while both paralogs contribute unequally to cranial development. The study provides a genetic framework for understanding how duplicated developmental genes evolve and how neural-crest-derived pigmentation and skeletal traits are coordinated in teleost fishes.

**Abstract:**

Neural crest cells give rise to the craniofacial skeleton and multiple pigment cell lineages, yet how duplicated developmental regulators partition their ancestral functions after teleost-specific whole-genome duplication remains unclear. Here, we employed CRISPR/Cas9 to generate *alx4a* and *alx4b* single and double mutants in Nile tilapia (*Oreochromis niloticus*). By integrating phenotype, skeleton, transcriptome, quantitative PCR, and AlphaFold-based structural modeling analyses, we revealed their functional divergence. Loss of *alx4a* caused a regionally restricted reduction in iridophore-derived reflectance and abnormal cranial morphology, whereas *alx4b* single mutants showed no obvious phenotype under the conditions examined. By contrast, double mutants exhibited an almost complete loss of iridophore-derived structural coloration and substantially more severe cranial defects, accompanied by reduced calcein labeling in the opercular region, consistent with altered cranial mineralization. Skin transcriptomic and quantitative PCR analyses revealed marked downregulation of *pnp4a* and *tfec*, which are associated with iridophore differentiation and coloration, whereas no significant expression differences were detected for the iridophore survival-related genes *ltk* and *mpv17*. AlphaFold2-assisted HDOCK protein–DNA modeling yielded more favorable docking metrics for Alx4a than for Alx4b with the *pnp4a* promoter, supporting a potential Alx4a–*pnp4a* promoter interaction that requires experimental validation. In contrast, no significant genotype-dependent differences were detected in the measured abundance of melanophores, xanthophores, or erythrophores, and no obvious difference in gross dorsal-fin spine formation was observed under the conditions examined. Together, these findings reveal unequal functional contributions of *alx4a* and *alx4b*, with *alx4a* acting as the dominant paralog in iridophore-associated structural coloration and both paralogs contributing unequally to cranial development, and support *pnp4a* as a candidate downstream gene associated with Alx4a activity.

## 1. Introduction

Teleost fishes possess the most diverse repertoire of pigment cells among vertebrates, including melanophores, xanthophores, erythrophores, iridophores, leucophores, and cyanophores [1,2,3,4]. Iridophores generate structural coloration through guanine-containing reflective platelets and contribute to body patterning, camouflage, and visual communication [5,6,7]. Like most of the craniofacial skeleton, they arise from neural crest cells (NCCs) [8,9]. Although these derivatives acquire distinct identities and functions, their common origin raises the possibility that aspects of their developmental regulation are shared.

The Alx family encodes conserved homeobox transcription factors characterized by a DNA-binding homeodomain and a C-terminal OAR domain [10]. In vertebrates, Alx proteins regulate neural-tube closure, appendage patterning, and craniofacial development [11,12], and most extant species retain three family members, Alx1, Alx3, and Alx4 [13]. Alx4 is particularly relevant to neural-crest-derived tissues as conditional deletion of Alx4 causes craniofacial and limb abnormalities in mice [14], and loss of *alx4a* in zebrafish disrupts cutaneous iridophore development [15]. These findings point to a conserved developmental role for Alx4, but they do not explain how that role has changed following gene duplication in teleosts.

The teleost-specific third round of whole-genome duplication generated two *alx4* paralogs, *alx4a* and *alx4b*. Their retention poses a central question in duplicate gene evolution of whether the two copies remained redundant, divided ancestral functions, or diverged asymmetrically as one paralog became functionally predominant. Previous work has focused largely on *alx4a* [14,15], leaving the contribution of *alx4b* unresolved. Because a retained paralog may act only in particular tissues or become evident only when the dominant copy is removed, single mutant analysis alone cannot distinguish redundancy from unequal functional divergence.

Nile tilapia (*Oreochromis niloticus*) provides a valuable model system to investigate the functional relationship between duplicated *alx4* paralogs. It is an established model for pigment-pattern formation and chromatophore biology [16,17,18], and it also possesses a readily accessible craniofacial skeleton and true dorsal fin spines. This provides an opportunity to evaluate whether duplicated *alx4* genes also influence acanthomorph-specific skeletal traits. The latter is relevant because *alx4a* has been implicated in fin-spine development in acanthomorph fishes [19], a possibility that cannot be tested directly in zebrafish, which lack true fin spines. Therefore, tilapia allows for the examination of pigmentary and skeletal phenotypes of *alx4* disruption in the same species.

Here, we generated *alx4a* and *alx4b* single and double mutants in Nile tilapia. By combining comparative genomic analysis with developmental, skeletal, and molecular phenotyping, we assessed the relative contributions of the two paralogs to iridophore and cranial development. Our results reveal unequal, tissue-dependent functional contributions of *alx4a* and *alx4b* and provide a genetic framework for investigating how duplicated developmental regulators diverge across neural-crest-derived traits in teleosts.

## 2. Materials and Methods

### 2.1. Animals

Wild-type Nile tilapia (*Oreochromis niloticus*) were maintained at 26 °C in a recirculating aquaculture system under a natural photoperiod. All animal procedures were conducted in accordance with the Guide for the Care and Use of Laboratory Animals and were approved by the Institutional Animal Care and Use Committee of Southwest University, China (approval no. IACUC-20181015-12, 15 October 2018).

### 2.2. Phylogenetic Analysis and Nucleotide Sequence Alignment

Human *ALX1*, *ALX3*, and *ALX4* sequences reported in the literature were used as queries for BLASTN searches of the National Center for Biotechnology Information (NCBI) database (https://www.ncbi.nlm.nih.gov/ (accessed on 19 August 2026)). *Alx1*, *Alx3*, and *Alx4* family sequences were retrieved, when present, from representative jawless vertebrates (*Myxine glutinosa* and *Petromyzon marinus*), cartilaginous fishes (*Callorhinchus milii* and *Carcharodon carcharias*), lobe-finned fishes (*Protopterus annectens*), ray-finned fishes (*Lepisosteus oculatus*, *Anguilla anguilla*, *Danio rerio*, *Ctenopharyngodon idella*, *Astyanax mexicanus*, *Myxocyprinus asiaticus*, *Ictalurus punctatus*, *Oryzias latipes*, *Oreochromis aureus*, *Oreochromis niloticus*, and *Takifugu rubripes*), amphibians (*Xenopus laevis*), reptiles (*Podarcis muralis*), birds (*Gallus gallus*), and mammals (*Mus musculus*).

Sequences were saved in FASTA format and imported into MEGA 12 (Version 12.0.11; MEGA Software, Institute for Genomics and Evolutionary Medicine, Temple University, Philadelphia, PA, USA; official website: https://www.megasoftware.net (accessed on 19 August 2026)). Multiple sequence alignment was performed using the ClustalW algorithm implemented in MEGA 12 with the default scoring and gap-penalty parameters. A neighbor-joining (NJ) phylogenetic tree was constructed from the aligned nucleotide sequences using the Kimura 2-parameter substitution model, with human *PAX7* as the outgroup. Branch support was evaluated using 1000 bootstrap replicates; the number of bootstrap replicates was manually set to 1000, whereas all other phylogenetic-analysis settings were kept at the MEGA 12 default values. The analysis was used to examine *Alx* gene copy number and evolutionary relationships across major vertebrate lineages. All sequences included in the analysis are listed in Table S1.

### 2.3. Chromosomal Synteny and Protein Sequence Analysis of Vertebrate Alx4

Chromosomal locations and neighboring genes surrounding Alx4 were obtained from NCBI and the Genomicus Genome Browser (https://www.genomicus.biologie.ens.fr/genomicus (accessed on 19 August 2026)) for representative jawless vertebrates, cartilaginous fishes, ray-finned fishes, lobe-finned fishes, and tetrapods. Syntenic relationships were compared to infer the origin and evolutionary history of vertebrate Alx4 loci.

Predicted Alx4 amino acid sequences were downloaded in FASTA format and subjected to multiple sequence alignment using the ClustalW algorithm implemented in MEGA 12 (Version 12.0.11) with the default protein scoring and gap-penalty parameters. Pairwise amino acid sequence similarities were subsequently calculated using MegAlign in the Lasergene v7.1 software package (DNASTAR, Inc., Madison, WI, USA), with particular attention to the conserved homeodomain and OAR domain.

### 2.4. Establishment of alx4a and alx4b Single- and Double-Mutant Lines Using CRISPR/Cas9

CRISPR/Cas9 target sites were designed using the CRISPRdirect online server (https://crispr.dbcls.jp/ (accessed on 19 August 2026)) in exon 3 of Nile tilapia *alx4a* and exon 1 of *alx4b*, as described previously. The *alx4a* target comprised the 20-nt protospacer 5′-GCGGCCTCCGATAGGACGCC-3′ followed by an AGG protospacer-adjacent motif (PAM), whereas the *alx4b* target comprised 5′-CCGGGTAAAGGGCAGGCTTA-3′ followed by a CGG PAM.

Guide RNAs (gRNAs) targeting *alx4a* and *alx4b* were synthesized in vitro using the gene-specific forward primers *alx4a*-gRNA-F and *alx4b*-gRNA-F, respectively, together with the common reverse primer gRNA-R. For simultaneous editing, the *alx4a* and *alx4b* gRNA stocks (1000 ng/µL each) were combined at a 1:1 volume ratio. The combined gRNA preparation (1000 ng/µL) was mixed at a 1:1 ratio (*v*/*v*) with Cas9 mRNA (1000 ng/µL), and phenol red was added to 10% of the final injection volume. The mixture was microinjected into one-cell-stage fertilized eggs. Surviving embryos were reared to sexual maturity. F0 founders were initially screened by locus-specific PCR amplification using the *alx4a*-page-F/*alx4a*-page-R and *alx4b*-page-F/*alx4b*-page-R primer pairs. Candidate mutant alleles were subsequently confirmed by Sanger sequencing using the *alx4a*-test-F/*alx4a*-test-R and *alx4b*-test-F/*alx4b*-test-R primer pairs. The sequences of all primers used for genotyping and Sanger sequencing are provided in the Supplementary Materials.

F0 mosaic males carrying confirmed mutations were crossed with wild-type (WT) XX females from the same Nile tilapia genetic background to generate F1 offspring. F1 progenies were genotyped using the same PCR amplification and Sanger-sequencing workflow, and individuals carrying the selected frameshift alleles were retained. F1 males and females carrying the same allele were intercrossed to establish homozygous F2 single-mutant lines. To generate the double-mutant line, F1 fish carrying both selected alleles were intercrossed, and F2 progeny homozygous at both loci were identified using the same PCR-based genotyping and Sanger-sequencing strategy. The final lines carried a 5-bp deletion in *alx4a* and a 7-bp deletion in *alx4b* (Figure S3).

All WT and mutant fish used in this study were derived from the same genetic background and maintained under identical husbandry conditions, with sibling WT fish used as controls. F0 founders were crossed with WT fish of the same genetic background during establishment of the F1 generation, and no additional backcrossing was performed. Potential off-target effects were minimized during gRNA design using CRISPRdirect. No genome-wide off-target sequencing was performed.

### 2.5. Phenotypic Analysis and Pigment Cell Counting

WT, *alx4a*^−/−^, *alx4b*^−/−^, and *alx4a*^−/−^;*alx4b*^−/−^ fish (*n* = 3 per genotype) were photographed at 5, 90, and 180 dpf. Fish younger than 10 dpf were imaged using a Leica M165 FC stereomicroscope (Leica Microsystems, Wetzlar, Germany), and juvenile or adult fish were photographed using a Nikon D7000 camera (Nikon, Tokyo, Japan). Body bars, interbars, caudal fins, and scales were examined to compare pigment-cell morphology and abundance among genotypes.

To improve melanophore visibility during cell counting, fish were anesthetized in 4.5 mg/mL of tricaine methanesulfonate (MS-222; Western Chemical Inc., Ferndale, WA, USA) and then immersed in 10 mg/mL of L-epinephrine hydrochloride (Sigma-Aldrich, St. Louis, MO, USA) for 15 min to induce pigment aggregation [20,21]. Fin pieces of approximately 25 mm^2^ were excised, mounted on glass slides, and imaged using an Olympus BX53 microscope (Olympus, Tokyo, Japan). Xanthophores were identified by their green fluorescence under 488-nm excitation in scale preparations, whereas erythrophores were identified by their red fluorescence under 561-nm excitation in caudal-fin preparations [22,23,24]. Xanthophore and erythrophore abundance was quantified as the number of fluorescence-positive cells within standardized microscopic fields of identical area. Cells were manually counted using ImageJ software (version 1.54f; National Institutes of Health, Bethesda, MD, USA) under identical imaging conditions across all genotypes. For each fish, three non-overlapping microscopic fields were analyzed, and the mean cell count from these fields was used as the value for that individual. Three individual fish were analyzed for each genotype (*n* = 3 biological replicates), with each fish, rather than each microscopic field, considered one independent biological replicate.

### 2.6. Quantitative Real-Time PCR

Total RNA was extracted from dorsal skin tissues of 90 dpf fish (*n* = 3) using RNAiso reagent (Takara, Kusatsu, Japan). RNA concentration was measured using a NanoDrop 2000 spectrophotometer (Thermo Fisher Scientific, Wilmington, DE, USA). Reverse transcription and cDNA synthesis were performed using the PrimeScript RT Reagent Kit with gDNA Eraser (Takara, Japan). The resulting cDNA was diluted tenfold and used as the quantitative real-time PCR (qPCR) template. Reactions were performed according to the manufacturer’s instructions for TB Green Premix Ex Taq II (Takara). The suitability of β-actin as the internal reference gene under the present experimental conditions was evaluated by semi-quantitative RT-PCR using the same dorsal skin samples subsequently used for qPCR analysis, including WT, *alx4a*^−/−^, *alx4b*^−/−^, and *alx4a*^−/−^;*alx4b*^−/−^ double-knockout fish (*n* = 3 independent biological replicates per genotype). Comparable β-actin amplification was observed across the 12 biological samples (Figure S4), supporting its use as the internal reference gene for qPCR normalization.

Relative expression was calculated using the 2^−ΔΔCt^ method, with β-actin as the internal reference gene. Gene-specific primer sequences are listed in Table S2.

### 2.7. RNA Sequencing and Bioinformatic Analysis

Dorsal skin samples were collected from 90 dpf wild-type (WT) and *alx4a*/*alx4b* double-mutant Nile tilapia, with three independent biological replicates per genotype. Total RNA was quality-checked before library construction. Poly(A)-containing mRNA was enriched, and RNA-seq libraries were prepared according to the standard transcriptome sequencing workflow of Tsingke Biotechnology Co., Ltd. (Beijing, China). Libraries were sequenced on the BGISEQ-500 platform using paired-end 150-bp sequencing.

Raw reads were filtered using fastp v0.20.0. Across the six libraries, 41.04 Gb of clean data was obtained, with 42.84–52.85 million clean reads per sample, Q30 values ≥ 97.91%, and effective data rates of 97.01–99.29%. Clean reads were aligned to the Nile tilapia reference genome O_niloticus_UMD_NMBU (NCBI RefSeq assembly GCF_001858045.2) and its corresponding RefSeq gene annotation using HISAT2 v2.2.1. Overall mapping rates ranged from 80.34% to 91.01%, and uniquely mapped reads accounted for 77.23%–86.91%.

Transcript abundance was quantified using StringTie v2.0.4. Differential expression analysis was performed using DESeq2 v1.26.0. DESeq2 output included both raw *p* values and Benjamini–Hochberg-adjusted *p* values (padj; false discovery rate, FDR). Differentially expressed genes (DEGs) were defined using only the FDR-adjusted significance criterion, with padj < 0.05 and |log2FC| ≥ 1. Raw *p* values were retained as intermediate statistical output but were not used for DEG selection, thereby reducing false-positive findings arising from multiple testing. Hierarchical clustering was performed using pheatmap v1.0.12, and GO and KEGG enrichment analyses were conducted using clusterProfiler v3.14.0. Unless otherwise specified, all bioinformatic software was run using default parameters. The raw and processed RNA-seq data have been deposited in the NCBI Gene Expression Omnibus (GEO) under accession number GSE343457.

### 2.8. Comparative Skeletal Analysis

For direct morphological comparison, healthy, size-matched WT and mutant adults at 180 dpf were photographed using a Nikon D7000 camera, and cranial morphology was compared among genotypes. For in vivo skeletal staining, healthy, size-matched WT and *alx4a*/*alx4b* double-mutant juveniles at 30 dpf were immersed in 0.2% calcein for 10 min, rinsed in clean water for 5 min, and subjected to three staining cycles in the dark [25]. Fluorescent skeletal mineralization was imaged using a Nikon SMZ25/SMZ18 stereomicroscope (Nikon Corporation, Tokyo, Japan).

### 2.9. AlphaFold-Based Prediction of Alx4–pnp4a Promoter Interactions

The *pnp4a* promoter fragment used for protein–DNA docking was retrieved from the Nile tilapia reference genome assembly O_niloticus_UMD_NMBU (RefSeq assembly accession: GCF_001858045.2). The promoter sequence was extracted from linkage group LG5 (RefSeq accession: NC_031970.2), spanning the genomic coordinates 11,896,276–11,898,276, which corresponds to a 2001-bp genomic fragment upstream of the annotated transcription start site of *pnp4a*. The nucleotide sequence was exported in FASTA format from the reference genome and served as the DNA input for molecular docking analysis. This fragment is located on the forward (+) strand of linkage group LG5.

The amino acid sequences of Nile tilapia Alx4a and Alx4b and the nucleotide sequence of the *pnp4a* promoter fragment were retrieved from the National Center for Biotechnology Information (NCBI) database in FASTA format. Three-dimensional structures of Alx4a and Alx4b were predicted using AlphaFold 2 (version 2.3.2, DeepMind, London, UK), and the top-ranked model for each paralog was retained for subsequent docking analysis.

Protein–DNA docking between the AlphaFold-predicted Alx4a or Alx4b structure and the modeled double-stranded *pnp4a* promoter fragment was subsequently performed using the HDOCK web server (Huang Laboratory, Huazhong University of Science and Technology, Wuhan, China). HDOCK employs a hybrid template-based/template-free strategy together with an FFT-based global docking search, and candidate binding modes are evaluated using its iterative knowledge-based scoring function. The entire promoter fragment analyzed in this study was used as the DNA docking partner. Docking was performed with the default HDOCK settings and without predefined binding-site restraints. Docking solutions were ranked according to the HDOCK docking score, with more negative values representing more favorable scores within the HDOCK scoring scheme, and the corresponding confidence score was recorded. For each Alx4 paralog, the top-ranked model with the most negative docking score was selected for subsequent interface analysis.

For analysis of the predicted protein–DNA interface, amino acid residues and promoter nucleotides within a maximum interatomic distance of 10 angstroms (Å) were considered potential interacting residues. Predicted hydrogen-bond and electrostatic contacts and their interatomic distances were subsequently examined and visualized using PyMOL, Version 2.6 (Schrödinger, LLC, New York, NY, USA). Because these analyses were entirely computational, HDOCK docking scores and confidence scores were interpreted as model-ranking metrics rather than experimentally measured binding affinities, and the predicted Alx4–*pnp4a* promoter interactions were treated as structural hypotheses rather than evidence of direct promoter binding or transcriptional regulation in vivo.

### 2.10. Statistical Analysis

All statistical analyses were performed using GraphPad Prism 8.0.1 (GraphPad Software, San Diego, CA, USA). All data were presented as the mean ± standard deviation (SD). Unless otherwise specified, *n* represents the number of independent biological replicates, with each biological replicate corresponding to an individual fish. Technical replicates obtained from the same biological sample were averaged before statistical analysis. Before parametric testing, data normality was assessed using the Shapiro–Wilk test, and homogeneity of variance was evaluated using the Brown–Forsythe test implemented in Prism. For comparisons between two groups, an unpaired two-tailed Student’s *t*-test was used when parametric assumptions were satisfied; Welch’s *t*-test was used when variances were unequal. For comparisons among three or more groups, ordinary one-way analysis of variance (ANOVA) followed by Tukey’s multiple-comparison test was used when normality and homogeneity-of-variance assumptions were satisfied. When data were approximately Gaussian but variances were unequal, Welch/Brown–Forsythe one-way ANOVA followed by Dunnett’s T3 multiple-comparison test was used. When the normality assumption was not met, nonparametric tests were used (Mann–Whitney test for two groups; Kruskal–Wallis test followed by Dunn’s multiple-comparison test for three or more groups). A *p* value < 0.05 was considered statistically significant.

## 3. Results

### 3.1. Phylogenetic Analysis Reveals Lineage-Specific Retention of Teleost alx4 Paralogs

**Figure 1:**
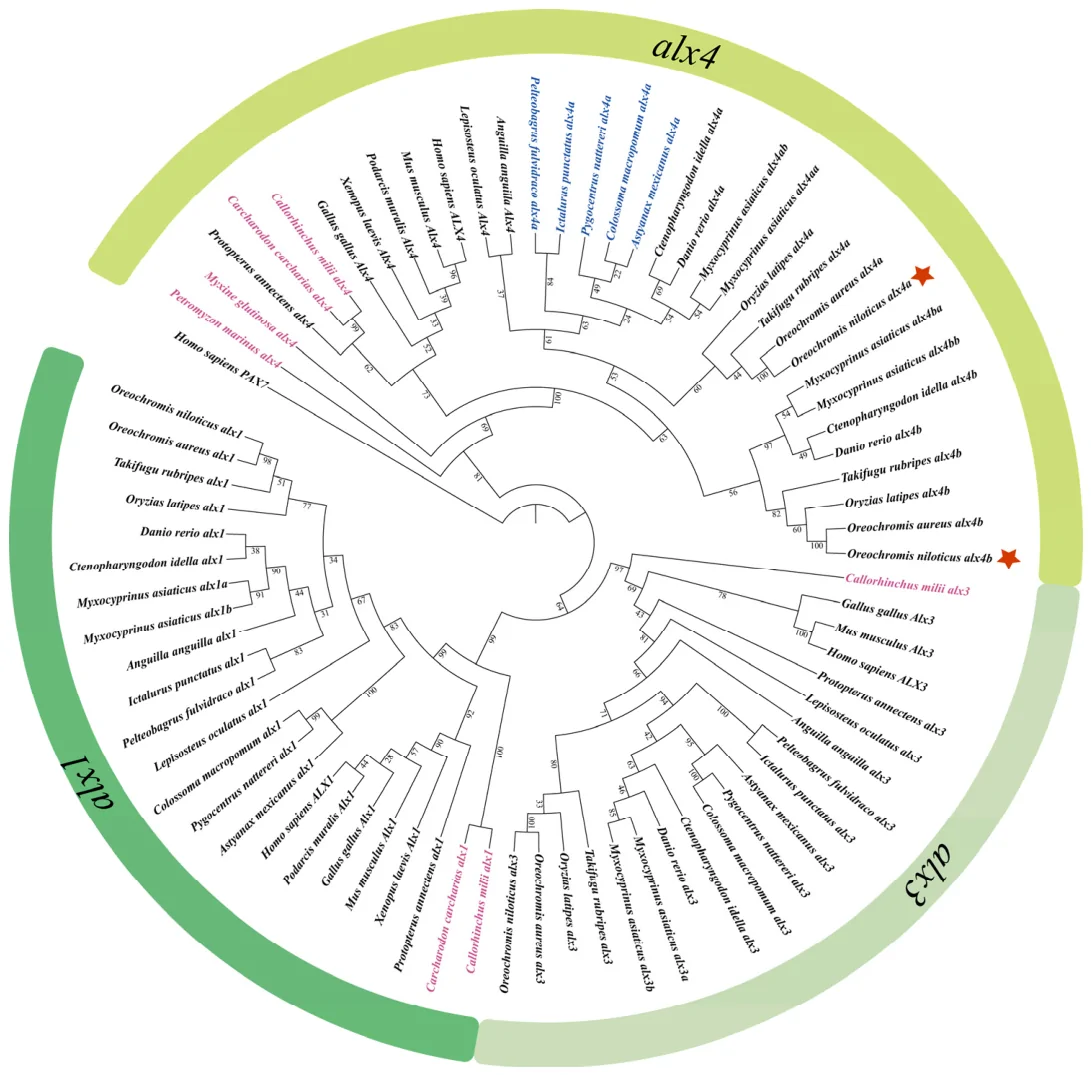
Phylogenetic relationships of the vertebrate *Alx* gene family. A neighbor-joining tree was constructed using human *PAX7* as the outgroup. Numbers at nodes indicate bootstrap support values from 1000 replicates. Red stars indicate *alx4a* and *alx4b* from Nile tilapia (*Oreochromis niloticus*). *Alx* family members from *Myxine glutinosa*, *Petromyzon marinus*, *Callorhinchus milii*, and *Carcharodon carcharias* are shown in pink. *alx4a* sequences from Siluriformes and Characiformes are shown in blue.

Phylogenetic reconstruction separated the vertebrate *Alx* family into three major clades corresponding to *Alx1*, *Alx3*, and *Alx4*, with *Alx1* and *Alx3* forming closely related clades (Figure 1). Among the sampled jawless vertebrates, only *Alx4*-like sequences were identified in the analyzed datasets, whereas cartilaginous fishes contained representatives of all three *Alx* clades. This distribution, together with the tree topology, is consistent with expansion of the ancestral *Alx* repertoire during the first two rounds of vertebrate whole-genome duplication, followed by the divergence of *Alx1* and *Alx3* as distinct paralogs.

Most teleost species retained both *alx4a* and *alx4b*, consistent with their origin in the teleost-specific whole-genome duplication. By contrast, only *alx4a* was identified in *Astyanax mexicanus* and *Ictalurus punctatus*. The same pattern was observed in additional representatives of Characiformes and Siluriformes, including *Colossoma macropomum*, *Pygocentrus nattereri*, and *Pelteobagrus fulvidraco*, supporting lineage-specific loss of Alx4b in these groups. The allotetraploid *Myxocyprinus asiaticus*, which underwent an additional genome duplication, retained four copies—*alx4aa*, *alx4ab*, *alx4ba*, and *alx4bb*. Together, these results support the hypothesis that *alx4a* and *alx4b* originated during the teleost-specific genome duplication and reveal that their subsequent retention has varied among teleost lineages.

### 3.2. Conserved Synteny and Protein Architecture of Vertebrate Alx4

A conserved genomic association between *alx4* and *tspan18* was identified in lamprey and across most of the sampled vertebrate lineages (Figure S1). This local syntenic relationship was not recovered in the examined cartilaginous fishes or lungfish, but remained evident in most ray-finned fishes and tetrapods, supporting long-term conservation of the *alx4* genomic neighborhood despite lineage-specific rearrangements.

Sequence comparisons revealed a similarly conserved protein architecture. All examined Alx4 proteins retained the characteristic homeodomain and OAR domain (Figure S2), and the lamprey sequences differed from their tetrapod counterparts at only a limited number of residues within these regions. The apparent gap in the predicted homeodomain of elephant shark Alx4 may reflect incomplete exon annotation rather than genuine domain loss. Both Nile tilapia paralogs shared more than 70% amino acid sequence similarity with Alx4 proteins from other bony fishes. Together, the conserved genomic context and domain organization indicate that both teleost Alx4 paralogs have remained under substantial evolutionary constraint following duplication.

### 3.3. alx4a Is the Predominant Paralog Contributing to Early Iridophore-Derived Reflectance

**Figure 2:**
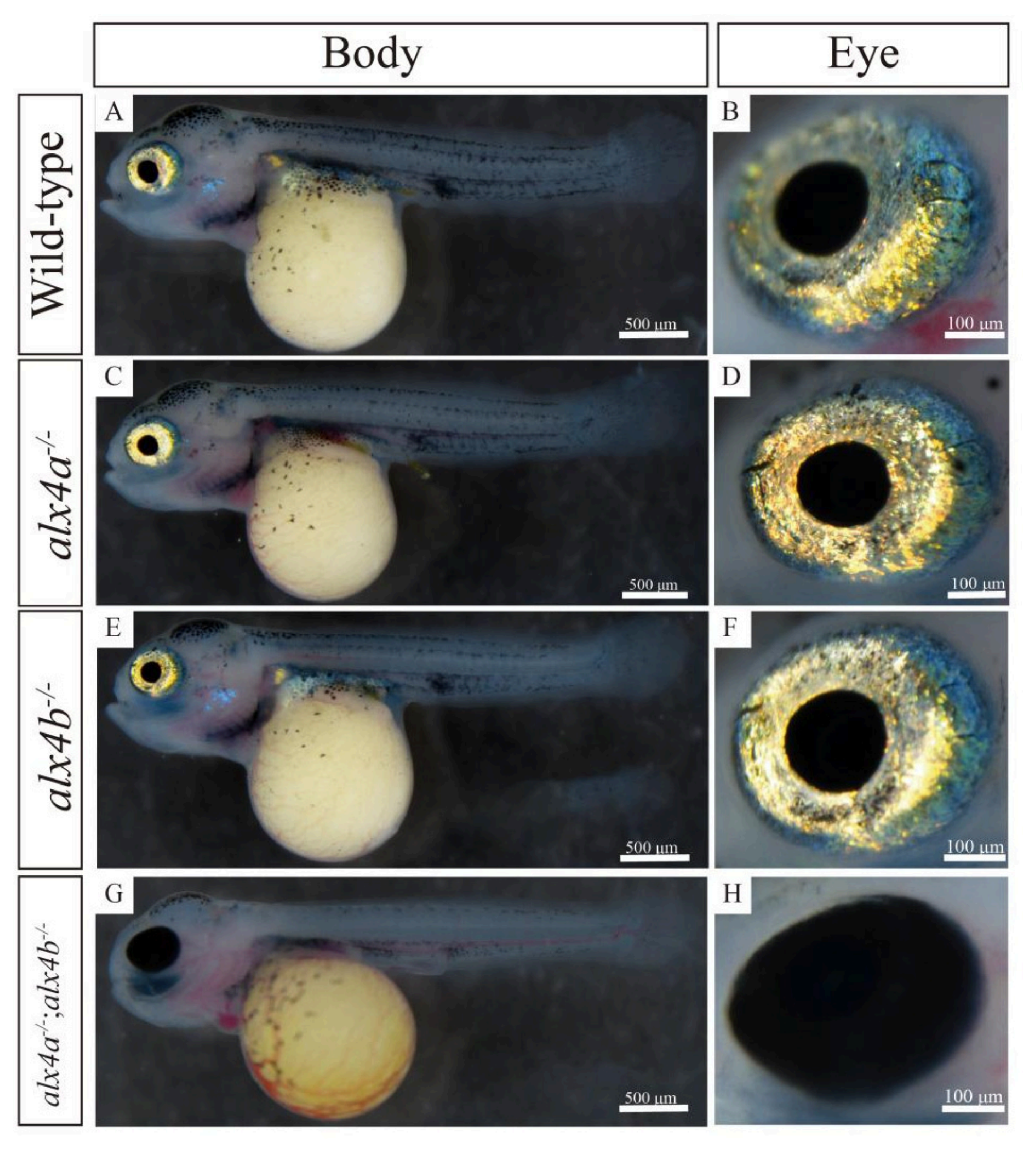
Phenotypes of WT and *alx4* mutant fish at 5 dpf. (**A**,**B**) Body and eye phenotypes of WT larvae. (**C**,**D**) Body and eye phenotypes of *alx4a*^−/−^ larvae. (**E**,**F**) Body and eye phenotypes of *alx4b*^−/−^ larvae. (**G**,**H**) Body and eye phenotypes of *alx4a*^−/−^;*alx4b*^−/−^ double mutants. Three individual fish were examined for each genotype (*n* = 3), and representative images are shown. dpf, days post-fertilization; WT, wild type.

At 5 days post-fertilization (dpf), no obvious difference in gross morphology was observed among WT and mutant larvae under the conditions examined, and melanophores were distributed over the head, trunk, and yolk-sac region in all four genotypes (Figure 2A,C,E,G). No obvious gross genotype-dependent difference in melanophore distribution or morphology was observed under the conditions examined. In WT larvae, iridophore-derived metallic reflectance was evident in the iris, opercular region, and peritoneum, and the iris displayed a conspicuous golden sheen (Figure 2A,B).

In *alx4a*^−/−^ larvae, the golden iris reflectance was largely retained, whereas reflective pigmentation in the opercular region and peritoneum was markedly reduced or absent (Figure 2C,D). No obvious difference in overall appearance was observed between *alx4b*^−/−^ and WT larvae under the conditions examined, with metallic luster preserved in all examined regions (Figure 2E,F).

The *alx4a*^−/−^;*alx4b*^−/−^ larvae exhibited the most severe phenotype. Iridophore-derived reflectance was markedly reduced over most of the body, producing a translucent appearance through which the heart and vasculature were readily visible (Figure 2G). The iris lacked the characteristic metallic luster ring and appeared uniformly dark (Figure 2H). These observations indicate that *alx4a* provides the major contribution to early iridophore-derived reflectance, whereas *alx4b* may contribute to residual iridophore-derived reflectance, particularly in the iris, when *alx4a* is absent.

### 3.4. alx4a and alx4b Contribute Unequally to Juvenile Iridophore-Derived Coloration

**Figure 3:**
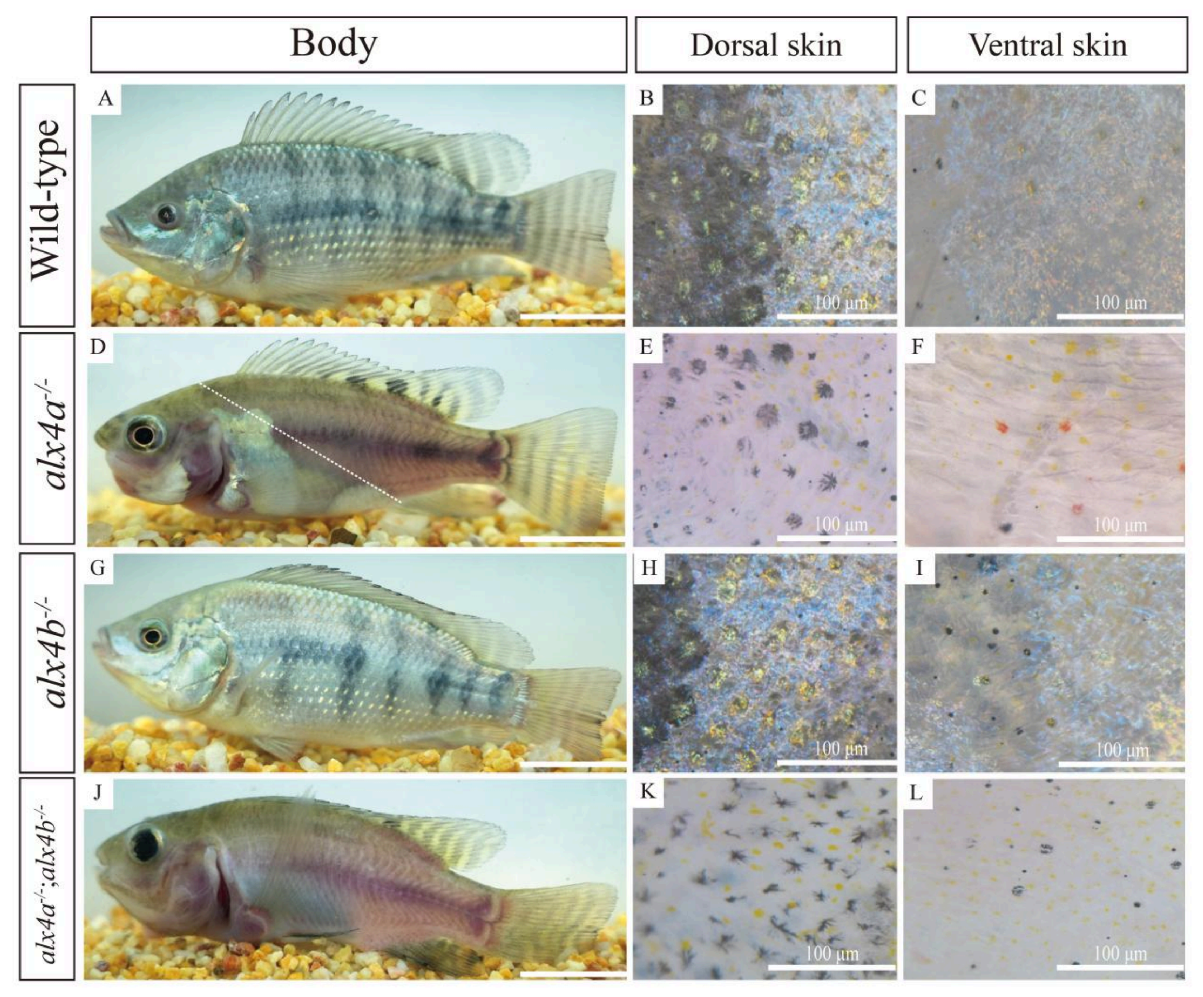
Phenotypes of WT and *alx4* mutant fish at 90 dpf. (**A**–**C**) Whole-body, dorsal-skin, and ventral-skin phenotypes of WT tilapia. (**D**–**F**) Corresponding phenotypes of *alx4a*^−/−^ fish. (**G**–**I**) Corresponding phenotypes of *alx4b*^−/−^ fish. (**J**–**L**) Corresponding phenotypes of *alx4a*^−/−^;*alx4b*^−/−^ double mutants. Three individual fish were examined for each genotype (*n* = 3), and representative images are shown. Scale bars in (**A**,**D**,**G**,**J**): 1 cm. dpf, days post-fertilization; WT, wild type.

At 90 dpf, WT juveniles displayed the characteristic tilapia pigmentation pattern, including well-defined vertical body bars and pigmented bands on the dorsal and caudal fins (Figure 3A). Dense iridophores in the dorsal and ventral skin contributed to a blue-gray metallic appearance (Figure 3B,C). *alx4a*^−/−^ juveniles retained iris reflectance and limited reflectance in the ventral skin, but the metallic coloration of the trunk, especially the dorsal skin, was markedly reduced, resulting in a pale body with less distinct bars (Figure 3D–F). No obvious difference in whole-body appearance or dorsal and ventral skin pigmentation was observed between *alx4b*^−/−^ and WT juveniles under the conditions examined (Figure 3G–I). In double mutants, reflective coloration was nearly absent from both dorsal and ventral skin, and the body appeared uniformly pale pink despite the persistence of melanophore-based dark elements (Figure 3J–L). The progressively stronger reduction from *alx4a* single mutants to double mutants further supports a dominant role for *alx4a* and a secondary, partially compensatory role for *alx4b* in juvenile iridophore coloration.

### 3.5. Adult alx4 Mutants Exhibit Iridophore-Selective Pigmentation Defects

**Figure 4:**
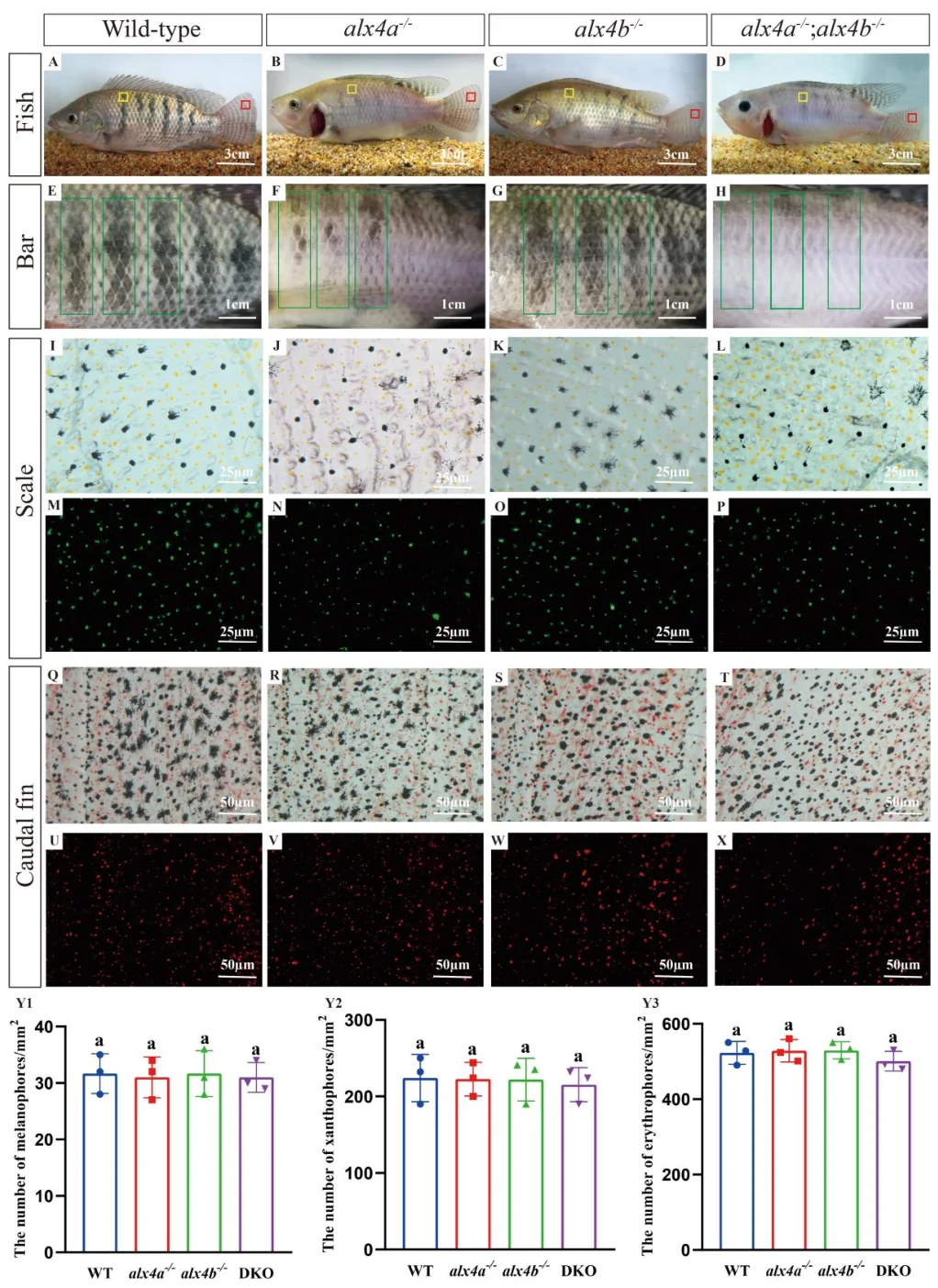
Adult pigmentation phenotypes of WT and *alx4* mutant tilapia at 180 dpf. (**A**–**D**) Whole-body phenotypes of WT, *alx4a*^−/−^, *alx4b*^−/−^, and *alx4a*^−/−^;*alx4b*^−/−^ fish. (**E**–**H**) Magnified body-bar patterns. (**I**–**L**) Scale preparations. (**M**–**P**) Fluorescence of xanthophores corresponding to the scales in (**I**–**L**). (**Q**–**T**) Caudal-fin preparations. (**U**–**X**) Fluorescence of erythrophores corresponding to the caudal fins in (**Q**–**T**). (**Y1**,**Y2**) Quantification of melanophores and xanthophores on scales. (**Y3**) Quantification of erythrophores in caudal fins. Three individual fish were analyzed for each genotype (*n* = 3 biological replicates). For each fish, three non-overlapping microscopic fields were analyzed and averaged to obtain one biological replicate. Data are presented as mean ± SD (*n* = 3). Statistical differences were assessed by one-way ANOVA followed by Tukey’s multiple-comparison test; same lowercase letters above the bars indicate no significant difference (*p* ≥ 0.05). Colored boxes indicate the sampled scale, caudal-fin, and bar regions. dpf, days post-fertilization; DKO, double knockout; WT, wild type.

At 180 dpf, WT fish displayed stable adult pigmentation, with continuous vertical bars and strong iridophore-derived reflectance over the body surface (Figure 4A,E). In *alx4a*^−/−^ adults, body reflectance remained strongly reduced, and the vertical bars were paler, narrower, and frequently discontinuous; however, the gross banding pattern of the dorsal and caudal fins was preserved (Figure 4B,F). No obvious difference in overall coloration or bar organization was observed between *alx4b*^−/−^ and WT adults under the conditions examined (Figure 4C,G). Double mutants retained the severe juvenile phenotype, including a pale pink body, extensive loss of metallic reflectance, and fragmentation of vertical bars into short segments or spots with poorly defined boundaries (Figure 4D,H). No obvious difference in fin banding was observed among genotypes under the conditions examined.

Scale and caudal fin samples were examined to determine whether the color phenotype reflected defects in other chromatophore lineages. Melanophores were readily observed on scales in all genotypes (Figure 4I–L). Xanthophores in the corresponding scale preparations emitted green fluorescence under 488-nm excitation (Figure 4M–P), and erythrophores in the caudal fin emitted red fluorescence under 561-nm excitation (Figure 4Q–X). Under the present experimental conditions, no significant genotype-dependent differences were detected in melanophore, xanthophore, or erythrophore abundance (Figure 4Y1–Y3). These results are consistent with the adult color phenotype being predominantly associated with iridophore-derived structural coloration rather than a generalized loss of other chromatophore classes.

### 3.6. Transcriptomic and qPCR Analyses Indicate Altered Expression of Genes Associated with Iridophore Differentiation Following alx4 Loss

**Figure 5:**
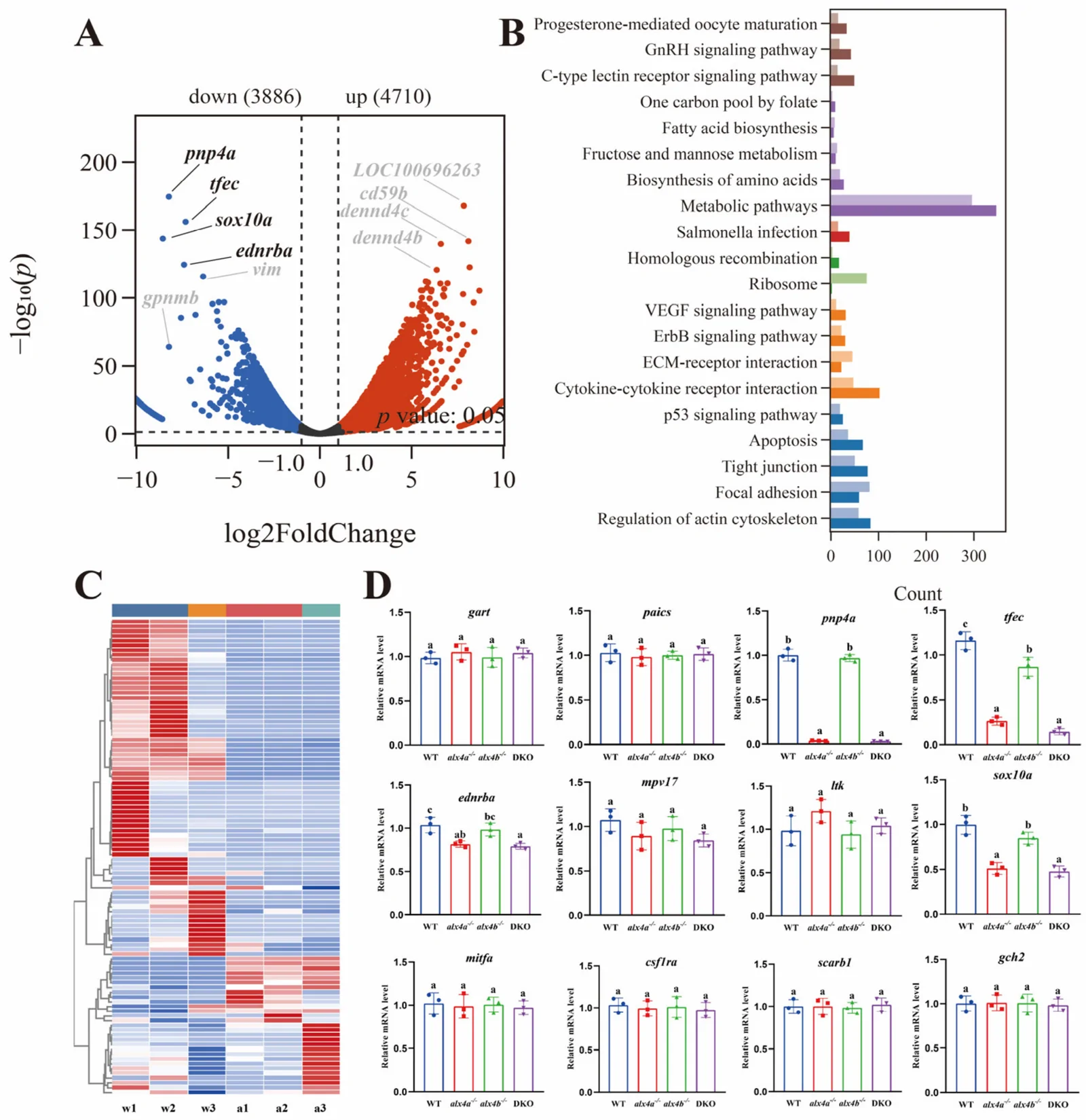
Skin transcriptome and qPCR analyses. (**A**) Volcano plot comparing dorsal skin from WT and *alx4a*^−/−^;*alx4b*^−/−^ fish at 90 dpf. RNA-seq was performed using three independent biological replicates per genotype (*n* = 3) Selected differentially expressed genes are labeled, with black labels indicating genes further validated by qPCR and grey labels indicating genes identified by RNA-seq but not subjected to qPCR validation. (**B**) KEGG enrichment analysis. (**C**) Hierarchical clustering heatmap of skin gene expression; colors from blue to red indicate low to high expression. (**D**) qPCR expression analysis of genes associated with the differentiation and coloration of melanophores, xanthophores, erythrophores, and iridophores in 90 dpf dorsal skin. Data are presented as mean ± SD (*n* = 3). Multiple-group comparisons were conducted by one-way ANOVA followed by Tukey’s test, and two-group comparisons by an unpaired two-tailed Student’s *t*-test. Different lowercase letters above the bars indicate significant differences (*p* < 0.05).

To identify molecular changes associated with the pigmentation phenotype, RNA sequencing was performed on dorsal skin from WT and *alx4a*^−/−^;*alx4b*^−/−^ fish at 90 dpf. Using the predefined FDR-based criteria (padj < 0.05 and |log2FC| ≥ 1), 4710 genes were upregulated and 3886 genes were downregulated in double mutants relative to WT controls (Figure 5A). Among the most strongly downregulated transcripts were the iridophore-associated genes *pnp4a*, *tfec*, *sox10a*, and *ednrba*, consistent with reduced iridophore-associated functions.

Kyoto Encyclopedia of Genes and Genomes (KEGG) enrichment analysis showed that the differentially expressed genes were distributed across multiple metabolic, signaling, and cellular-process pathways, with metabolic pathways containing the largest number of genes (Figure 5B). These results indicate that *alx4* loss is accompanied by broad transcriptional remodeling in the skin in addition to changes in pigment-cell regulators.

Hierarchical clustering of the transcriptomic profiles clearly separated WT and double-mutant samples, while biological replicates within each genotype showed similar expression patterns (Figure 5C), supporting the reproducibility of the RNA-sequencing dataset.

qPCR analysis was subsequently used to evaluate selected chromatophore-related genes across all four genotypes (Figure 5D). The iridophore differentiation and coloration genes *pnp4a*, *tfec*, and *sox10a* were strongly reduced in *alx4a*^−/−^ and double-mutant skin. *tfec* was also moderately but significantly reduced in *alx4b*^−/−^ skin, whereas no significant expression difference was detected for *pnp4a* or *sox10a* between *alx4b*^−/−^ and WT fish under the present experimental conditions. *ednrba* showed a weaker genotype-dependent decrease, with the lowest expression in double mutants. By contrast, under the present experimental conditions, no significant expression differences were detected for *gart*, *paics*, *mpv17*, *ltk*, *mitfa*, *csf1ra*, *scarb1*, or *gch2* among the examined genotypes. Together, the transcriptomic and qPCR data are consistent with altered expression of genes associated with iridophore differentiation and functional maturation following *alx4* loss, whereas no significant changes were detected in the examined markers of other chromatophore lineages under the present experimental conditions. However, the absence of significant changes in *mpv17* and *ltk* alone does not exclude possible effects on iridophore survival, maintenance, or cell viability.

### 3.7. Differential Effects of alx4 Mutations on Cranial Morphology and Opercular Mineralization

**Figure 6:**
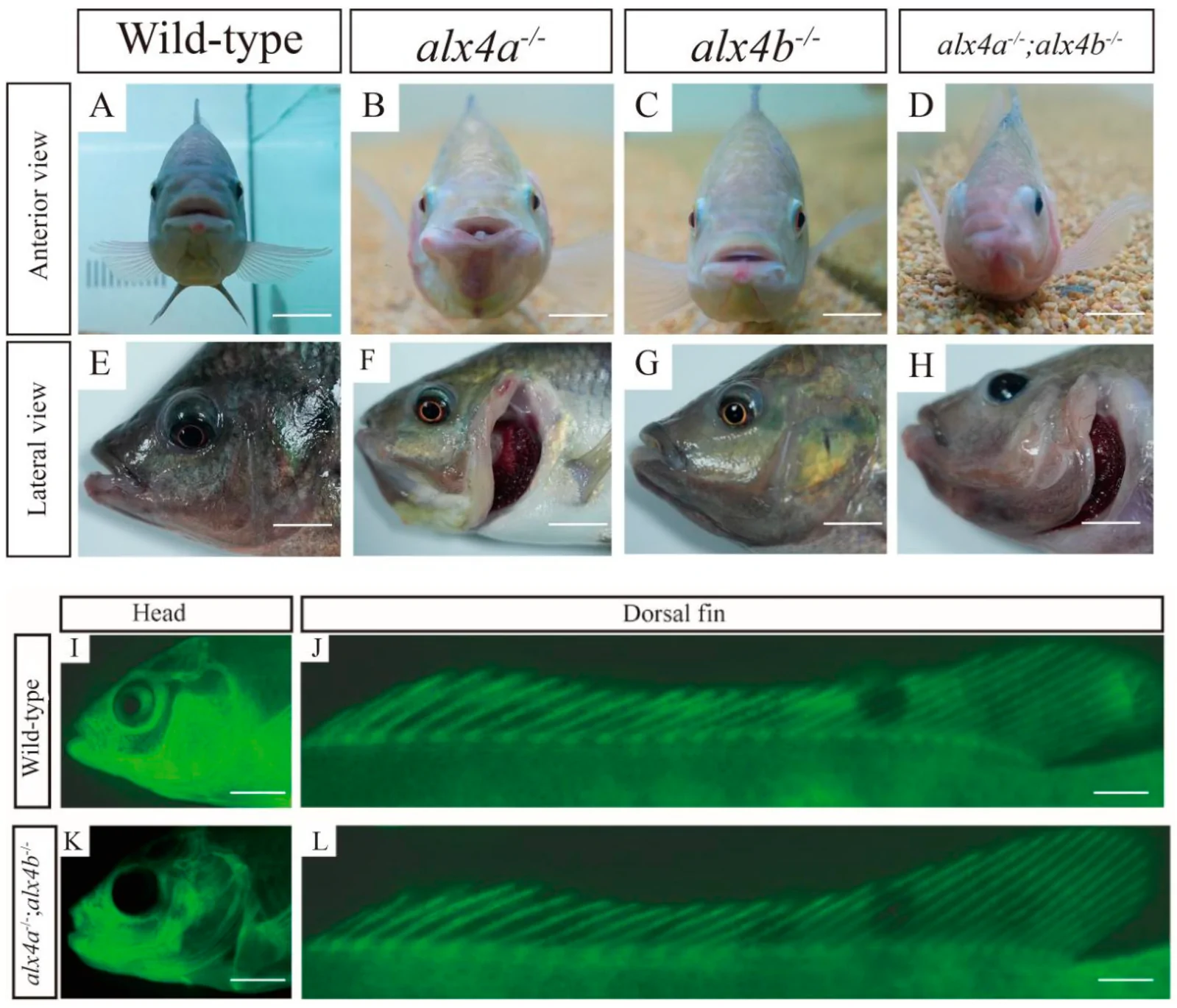
Cranial morphology across *alx4* genotypes and qualitative skeletal staining of WT and double-mutant tilapia. (**A**,**E**) Frontal and lateral views of the WT head. (**B**,**F**) Frontal and lateral views of the *alx4a*^−/−^ head. (**C**,**G**) Frontal and lateral views of the *alx4b*^−/−^ head. (**D**,**H**) Frontal and lateral views of the *alx4a*^−/−^;*alx4b*^−/−^ head. (**I**,**J**) Calcein staining of the WT head and dorsal fin. (**K**,**L**) Calcein staining of the double-mutant head and dorsal fin. Three individual fish were examined for each genotype for gross cranial morphology (*n* = 3 biological replicates). Calcein staining was evaluated qualitatively in three WT and three *alx4a*^−/−^;*alx4b*^−/−^ double-mutant fish (*n* = 3 biological replicates per genotype). Representative images are shown. Scale bars: 1 cm in (**A**–**H**) and 0.5 cm in (**I**–**L**).

Loss of *alx4* affected cranial morphology in a paralog-dependent manner. WT fish displayed the typical tilapia cranial architecture, with a relatively broad ventral head region, a slightly projecting snout, prominent eyes, and complete opercular coverage of the gill chamber (Figure 6A,E). In *alx4a*^−/−^ fish, the lower jaw was broadened and malformed, the snout was recessed, and the operculum showed incomplete coverage of the gill chamber (Figure 6B,F). By contrast, no obvious difference in gross cranial morphology was observed between *alx4b*^−/−^ and WT fish under the conditions examined (Figure 6C,G). Double mutants exhibited substantially more severe and asymmetric cranial abnormalities, including a conical head profile, recessed eyes, and incomplete opercular coverage (Figure 6D,H). These genotype-dependent phenotypes indicate that *alx4a* contributes predominantly to cranial development, whereas the additional defects in double mutants reveal a contribution from *alx4b*.

Qualitative calcein staining revealed differences in cranial calcein labeling between WT and double-mutant fish. WT fish showed strong fluorescence throughout the head and dorsal fin, consistent with active mineral deposition (Figure 6I,J). In double mutants, calcein labeling was markedly reduced in the opercular region, suggesting reduced mineral deposition in the region (Figure 6K). In contrast, the dorsal fin, including the fin spines, retained strong calcein labeling and no obvious gross reduction in dorsal-fin calcein labeling was observed under the conditions examined (Figure 6L). Because calcein staining was not quantitatively analyzed and comparable staining of the two single-mutant genotypes was not included, these observations do not establish the individual contributions of *alx4a* and *alx4b* to cranial mineralization or distinguish additive from cooperative genetic effects. Rather, they provide qualitative evidence that simultaneous loss of both paralogs is associated with altered opercular mineralization.

### 3.8. Structural Modeling Predicts More Favorable Docking of Alx4a with the pnp4a Promoter

**Figure 7:**
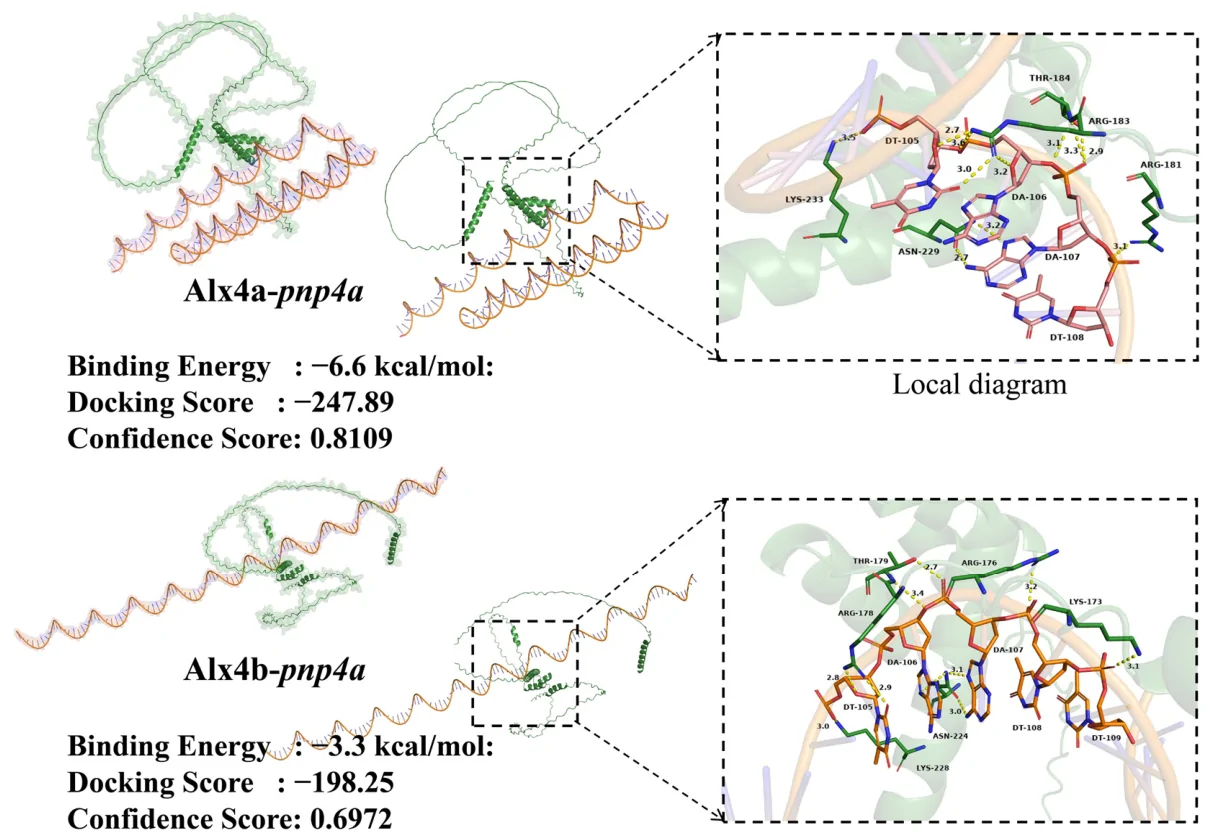
AlphaFold2-assisted structural modeling and HDOCK protein–DNA docking of Alx4a and Alx4b with the *pnp4a* promoter fragment. The top-ranked Alx4a complex had a docking score of −247.89 and a confidence score of 0.8109, whereas the top-ranked Alx4b complex had a docking score of −198.25 and a confidence score of 0.6972. Enlarged views show predicted interfacial contacts between Alx4 residues and promoter nucleotides. Dashed lines indicate predicted hydrogen-bond or electrostatic contacts, with distances shown in angstroms (Å). These computational models and HDOCK scores represent predicted docking solutions and do not experimentally demonstrate direct promoter binding, binding affinity, or transcriptional regulation. Protein–DNA docking was performed using the 2001-bp *pnp4a* promoter fragment from NC_031970.2 (11,896,276–11,898,276) with the default HDOCK settings and without predefined binding-site restraints.

AlphaFold2-assisted structural modeling followed by protein–DNA docking predicted potential interactions of both Alx4a and Alx4b with the *pnp4a* promoter fragment, with the Alx4a complex showing more favorable HDOCK docking metrics (Figure 7). The top-ranked Alx4a–*pnp4a* promoter complex had a docking score of −247.89 and a confidence score of 0.8109, whereas the top-ranked Alx4b–*pnp4a* promoter complex had a docking score of −198.25 and a confidence score of 0.6972. Because the HDOCK docking score is a model-ranking score rather than an experimentally calibrated binding free energy, these values were interpreted as indicating a more favorable predicted docking model for Alx4a and not as quantitative binding affinity.

Interface analysis of the Alx4a complex model identified Thr184, Arg181, Arg183, Asn229, and Lys233 as predicted interfacial residues. These residues formed multiple predicted hydrogen-bond or electrostatic contacts with nucleotides in the modeled promoter fragment, including DT105, DA106, DA107, and DT108, with interaction distances of approximately 2.7–3.5 Å. In the Alx4b complex, predicted contacts involved Lys173, Arg176, Arg178, Thr179, Asn224, and Lys228 and nucleotides spanning approximately DT105 to DT109; these structural differences were consistent with the comparatively less favorable docking metrics of the Alx4b complex.

Together with the pronounced reduction in *pnp4a* transcript abundance in *alx4a*^−/−^ and double-mutant skin, these in silico findings support *pnp4a* as a candidate downstream gene associated with Alx4a activity and suggest the possibility of an interaction between Alx4a and the *pnp4a* promoter. However, the computational analysis does not establish direct promoter binding or transcriptional regulation. Direct promoter occupancy and regulatory activity will require experimental validation, for example by chromatin immunoprecipitation, electrophoretic mobility-shift assays, or promoter-reporter assays.

## 4. Discussion

Comparison of the complete mutant series reveals an unequal functional relationship between the teleost *alx4* paralogs. Loss of *alx4a* alone disrupted iridophore-derived coloration and cranial development, whereas no obvious gross pigmentary or cranial abnormalities were observed in *alx4b* single mutants under the conditions examined. The substantially broader pigmentary defects and more severe cranial abnormalities in double mutants nevertheless demonstrate that *alx4b* is not functionally silent. Rather, *alx4a* makes the predominant contribution to both developmental processes, while *alx4b* provides a more limited contribution that becomes evident when *alx4a* is absent. This relationship was anatomically selective: no significant genotype-dependent differences were detected in the measured abundance of melanophores, xanthophores, or erythrophores or in the examined lineage-associated marker genes, whereas no obvious difference in gross dorsal-fin spine formation was observed under the conditions examined. Together, these findings support asymmetric, tissue-dependent functional divergence between the duplicated *alx4* paralogs in Nile tilapia.

### 4.1. Asymmetric Contributions of Duplicated alx4 Genes to Iridophore-Associated Structural Coloration

Iridophores generate structural coloration through intracellular reflective platelets containing ordered guanine crystals and contribute substantially to fish coloration and pattern formation [5,6,7]. Previous work in zebrafish implicated *alx4a* in iridophore development [9,15,26], but the absence of a corresponding double-mutant model left the contribution of *alx4b* unresolved. In Nile tilapia, loss of *alx4a* reduced iridophore-derived reflectance in defined anatomical regions, whereas simultaneous disruption of *alx4a* and *alx4b* produced a substantially broader phenotype. The increased severity of the double mutant shows that *alx4b* is not functionally silent, although no obvious phenotype was observed in *alx4b* single mutants under the conditions examined.

These genotype-dependent effects define an asymmetric relationship between the two paralogs. *alx4a* makes the predominant contribution to iridophore-associated reflective coloration, whereas *alx4b* provides a more limited contribution that becomes evident when *alx4a* is absent. The paralogs are therefore neither functionally equivalent nor completely redundant. This pattern is consistent with asymmetric functional divergence after duplication and may represent an asymmetric subfunctionalization scenario. However, the current genetic evidence does not formally distinguish asymmetric subfunctionalization from unequal functional contribution or partial functional redundancy. Distinguishing between these evolutionary scenarios will require comparative spatiotemporal expression analyses, reciprocal rescue experiments, and functional complementation assays to determine whether the phenotypic asymmetry arises from differences in regulatory activity, protein function or dosage, or partitioning of ancestral functions.

The molecular data further suggest that Alx4 activity is associated primarily with iridophore differentiation and reflective function rather than with a general failure of lineage maintenance. The marked reduction in *tfec* and *pnp4a* [27], together with the finding that no significant expression difference was detected for *ltk* or *mpv17* [28,29], is consistent with impaired differentiation, maturation, or guanine-dependent coloration. However, the absence of significant expression differences in *ltk* and *mpv17* does not exclude changes in iridophore abundance or viability. Accordingly, the visible loss of metallic reflectance should not be interpreted as definitive evidence that iridophores are completely absent; cells may persist but fail to acquire the organelles and guanine-rich platelets required for optical reflectance. Because iridophore number, density, and occupied area were not directly quantified in the present study, reduced reflectance alone cannot distinguish changes in iridophore abundance from defects in guanine crystal organization, reflective platelet formation, cellular maturation, or other optical properties. Therefore, our phenotypic and molecular data should be interpreted as being consistent with impaired iridophore differentiation and functional maturation rather than as direct evidence of defective differentiation. The more favorable HDOCK docking metrics for Alx4a than for Alx4b at the *pnp4a* promoter, together with the reduction in *pnp4a* expression in *alx4a* mutants, support *pnp4a* as a candidate downstream gene associated with Alx4 activity and suggest a potential Alx4a–*pnp4a* promoter interaction. Direct promoter occupancy and transcriptional regulation remain to be established experimentally.

### 4.2. Effects of alx4 Loss Are Predominantly Associated with the Iridophore Lineage

Despite the common neural crest origin of teleost chromatophores [5], no significant genotype-dependent differences were detected in the abundance of melanophores, xanthophores, or erythrophores or in the expression of the examined marker genes associated with these chromatophore lineages. By contrast, the most pronounced transcriptional changes involved the iridophore-associated genes *tfec* and *pnp4a*. This lineage-selective response argues against Alx4 acting as a general regulator of an early multipotent pigment-cell progenitor. Instead, its principal role appears to lie within the iridophore developmental program, particularly during the acquisition of reflective function.

The preservation of other chromatophore lineages also clarifies the origin of the visible pigmentation defects. The altered body color of *alx4* mutants is unlikely to result from a global disruption of chromatophore development, but rather from the loss of iridophore-derived reflectance against an otherwise largely preserved pigment-cell background. This interpretation is consistent with the retention of dark pigment cells and with the finding that no significant genotype-dependent differences were detected in melanophore, xanthophore, or erythrophore abundance.

Although the vertical body bars became paler and fragmented in *alx4* mutants, their basic spatial arrangement was retained. Alx4 is therefore unlikely to establish the primary positional framework of the bar pattern. Instead, iridophores appear to contribute to its continuity, contrast, and visual definition through interactions with other chromatophore classes. The mutant phenotype is thus consistent with a model in which the gross pattern is maintained by melanophores and other pigment cells, whereas Alx4-associated iridophore function contributes to refining the final appearance of the adult color pattern [30,31,32].

### 4.3. Alx4 Paralogs Contribute Unequally to Cranial Morphology

Alx4 has been implicated in vertebrate craniofacial development, with loss of *Alx4* in mice causing cranial and appendicular abnormalities [14]. By contrast, obvious cranial defects have not been reported in zebrafish carrying mutations in either *alx4a* or *alx4b* alone [8]. Our findings in Nile tilapia extend this comparative framework. Loss of *alx4a* altered jaw and opercular morphology, whereas simultaneous disruption of both paralogs produced more severe and asymmetric cranial abnormalities. The enhanced double-mutant phenotype indicates that *alx4b* contributes to cranial development, although no obvious cranial phenotype was observed in *alx4b* single mutants under the conditions examined. Alx4 function in the craniofacial skeleton may therefore be broadly conserved among vertebrates, while its phenotypic expression depends on paralog usage, species-specific regulatory context, and genetic background.

Reduced calcein labeling in the opercular region of double mutants is consistent with altered or reduced mineralization in this region. However, because calcein staining was assessed qualitatively and comparable staining data were not obtained for both single-mutant genotypes, the present analysis cannot determine the individual contributions of *alx4a* and *alx4b* to mineralization or establish whether their combined effects are additive, cooperative, or otherwise genetically interactive. Quantitative analyses of cranial dimensions, mineralized area, and calcein fluorescence, together with skeletal staining across all four genotypes, will be required to resolve these possibilities. The available evidence therefore supports a role for *alx4* paralogs in cranial morphogenesis and suggests an association between combined *alx4* loss and altered opercular mineralization, but does not yet define the underlying osteogenic mechanism.

The preservation of dorsal-fin spines provides an informative anatomical contrast. Although *alx4a* has been proposed to participate in fin-spine development in acanthomorph fishes [9], double-mutant tilapia retained recognizable spines with strong calcein labeling. Accordingly, no obvious difference in gross dorsal-fin spine formation or calcein labeling was observed under the conditions examined. Subtle effects on spine geometry, internal organization, mechanical properties, or earlier patterning cannot be excluded. Together, these results indicate that the skeletal consequences of *alx4* loss are regionally selective, with a substantially stronger requirement in the craniofacial skeleton than in the dorsal-fin spines of Nile tilapia.

## 5. Conclusions

We established a double-mutant genetic model for the duplicated tilapia genes *alx4a* and *alx4b* and revealed tissue-dependent functional divergence during the development of neural-crest-derived structures. *alx4a* is the major functional copy controlling iridophore structural coloration and cranial morphology, whereas no obvious gross pigmentary or cranial phenotype was observed in *alx4b* single mutants under the conditions examined, although *alx4b* contributes when *alx4a* is absent. Genetic and transcriptional evidence indicates that Alx4 primarily affects iridophore-associated structural coloration and is consistent with a role in iridophore differentiation and functional maturation, rather than broadly affecting other chromatophore lineages or dorsal-fin spine formation. Together, these findings link the unequal functional contributions of *alx4a* and *alx4b* to iridophore-associated structural coloration, cranial morphology, and altered opercular mineralization, and support a model of asymmetric functional divergence between the duplicated paralogs across distinct neural-crest-derived tissues.

## Data Availability

The RNA-seq dataset generated in this study has been deposited in the Gene Expression Omnibus (GEO) database under accession number GSE343457 (https://www.ncbi.nlm.nih.gov/geo/query/acc.cgi?acc=GSE343457 (accessed on 19 August 2026)). Other data supporting the findings of this study are available from the corresponding author upon reasonable request.

## Supplementary Materials

The following supporting information can be downloaded at: https://www.mdpi.com/article/10.3390/cells15171512/s1, Figure S1, Chromosomal synteny of Alx4 in vertebrates; Figure S2, Comparison of vertebrate Alx4 amino acid sequences; Figure S3, Establishment of the *alx4a* and *alx4b* single-mutant and double-mutant lines; Figure S4, Evaluation of β-actin expression in dorsal skin samples used for qPCR analysis. Table S1, Vertebrate Alx gene family members used for phylogenetic analysis; Table S2, Primers used for gRNA synthesis, genotyping, Sanger sequencing, and quantitative real-time PCR.
