# Supplementary material for: Asymmetric Functional Divergence of alx4a and alx4b in Iridophore Differentiation and Cranial Development in Nile Tilapia
Source: Cells. 2026 Aug 22;15(17):1512. doi: 10.3390/cells15171512 (PMC13564871; doi:10.3390/cells15171512)
Supplement: Supplementary file 1 [file cells-15-01512-s001.zip › Supplementary Materials.pdf]

Supplementary Materials

Asymmetric Subfunctionalization of *alx4a* and *alx4b* Regulates Iridophore Differentiation and Cranial Development in Nile Tilapia

Table S1. Vertebrate *alx* gene family members used for phylogenetic analysis.

Common name	Scientific name	Gene	NCBI accession number
Atlantic hagfish	*Myxine glutinosa*	*alx4*	XM_068123896.1
Sea lamprey	*Petromyzon marinus*	*alx4*	XM_032948818.1
Great white shark	*Carcharodon carcharias*	*alx1*	XM_041216282.1
Great white shark	*Carcharodon carcharias*	*alx4*	XM_041196925.1
Elephant shark	*Callorhinchus milii*	*alx1*	XM_007903776.2
Elephant shark	*Callorhinchus milii*	*alx3*	XM_042342236.1
Elephant shark	*Callorhinchus milii*	*alx4*	XM_042341374.1
West African lungfish	*Protopterus annectens*	*alx1*	XM_044087131.1
West African lungfish	*Protopterus annectens*	*alx3*	XM_044076907.1
West African lungfish	*Protopterus annectens*	*alx4*	XM_044078313.1
African clawed frog	*Xenopus laevis*	*Alx1*	XM_041588642.1
African clawed frog	*Xenopus laevis*	*Alx4*	XM_018257309.2
Common wall lizard	*Podarcis muralis*	*Alx1*	XM_028746065.1
Common wall lizard	*Podarcis muralis*	*Alx4*	XM_028727633.1
Red junglefowl	*Gallus gallus*	*Alx1*	XM_025154568.3
Red junglefowl	*Gallus gallus*	*Alx3*	XM_015299156.3
Red junglefowl	*Gallus gallus*	*Alx4*	NM_204162.2
House mouse	*Mus musculus*	*Alx1*	NM_001421475.1
House mouse	*Mus musculus*	*Alx3*	NM_007441.3
House mouse	*Mus musculus*	*Alx4*	NM_007442.3
Human	*Homo sapiens*	*ALX1*	NM_006982.3
Human	*Homo sapiens*	*ALX3*	NM_006492.3
Human	*Homo sapiens*	*ALX4*	NM_021926.4
Blue tilapia	*Oreochromis aureus*	*alx4b*	XM_039615790.1
Nile tilapia	*Oreochromis niloticus*	*alx1*	XM_003449158.5
Nile tilapia	*Oreochromis niloticus*	*alx3*	XM_025901873.1
Nile tilapia	*Oreochromis niloticus*	*alx4a*	XM_005466978.4
Nile tilapia	*Oreochromis niloticus*	*alx4b*	XM_003443874.5
Tiger pufferfish	*Takifugu rubripes*	*alx1*	XM_003967766.3
Tiger pufferfish	*Takifugu rubripes*	*alx3*	XM_003963208.2
Tiger pufferfish	*Takifugu rubripes*	*alx4a*	XM_011610177.2

Common name	Scientific name	Gene	NCBI accession number
Tiger pufferfish	*Takifugu rubripes*	*alx4b*	XM_003967522.3
Yellow catfish	*Pelteobagrus fulvidraco*	*alx1*	XM_027161241.2
Yellow catfish	*Pelteobagrus fulvidraco*	*alx3*	XM_027167441.2
Yellow catfish	*Pelteobagrus fulvidraco*	*alx4a*	XM_027178465.2
Tambaqui	*Colossoma macropomum*	*alx1*	XM_036585201.1
Tambaqui	*Colossoma macropomum*	*alx3*	XM_036578065.1
Tambaqui	*Colossoma macropomum*	*alx4a*	XM_036587261.1
Red-bellied piranha	*Pygocentrus nattereri*	*alx1*	XM_017695149.2
Red-bellied piranha	*Pygocentrus nattereri*	*alx3*	XM_017722036.1
Red-bellied piranha	*Pygocentrus nattereri*	*alx4a*	XM_017700676.2
Spotted gar	*Lepisosteus oculatus*	*alx1*	XM_006633549.3
Spotted gar	*Lepisosteus oculatus*	*alx3*	XM_015342431.2
Spotted gar	*Lepisosteus oculatus*	*alx4*	XM_006642480.3
European eel	*Anguilla anguilla*	*alx1*	XM_035427232.1
European eel	*Anguilla anguilla*	*alx3*	XM_035387527.1
European eel	*Anguilla anguilla*	*alx4*	XM_035418961.1
Zebrafish	*Danio rerio*	*alx1*	NM_001045074.1
Zebrafish	*Danio rerio*	*alx3*	XM_005167111.5
Zebrafish	*Danio rerio*	*alx4a*	XM_001340930.7
Zebrafish	*Danio rerio*	*alx4b*	NM_001310078.2
Grass carp	*Ctenopharyngodon idella*	*alx1*	XM_051870978.1
Grass carp	*Ctenopharyngodon idella*	*alx3*	XM_051903268.1
Grass carp	*Ctenopharyngodon idella*	*alx4a*	XM_051901147.1
Grass carp	*Ctenopharyngodon idella*	*alx4b*	XM_051871312.1
Mexican tetra	*Astyanax mexicanus*	*alx1*	XM_007249249.4
Mexican tetra	*Astyanax mexicanus*	*alx3*	XM_022671050.2
Mexican tetra	*Astyanax mexicanus*	*alx4a*	XM_007232898.3
Channel catfish	*Ictalurus punctatus*	*alx1*	XM_017465141.3
Channel catfish	*Ictalurus punctatus*	*alx3*	XM_017467405.3
Channel catfish	*Ictalurus punctatus*	*alx4a*	XM_017478917.3
Chinese sucker	*Myxocyprinus asiaticus*	*alx1a*	XM_051725057.1
Chinese sucker	*Myxocyprinus asiaticus*	*alx1b*	XM_051657249.1
Chinese sucker	*Myxocyprinus asiaticus*	*alx3a*	XM_051653386.1
Chinese sucker	*Myxocyprinus asiaticus*	*alx3b*	XM_051669330.1
Chinese sucker	*Myxocyprinus asiaticus*	*alx4aa*	XM_051700965.1

Common name	Scientific name	Gene	NCBI accession number
Chinese sucker	*Myxocyprinus asiaticus*	*alx4ab*	XM_051722314.1
Chinese sucker	*Myxocyprinus asiaticus*	*alx4ba*	XM_051724398.1
Chinese sucker	*Myxocyprinus asiaticus*	*alx4bb*	XM_051656388.1
Medaka	*Oryzias latipes*	*alx1*	XM_004079561.4
Medaka	*Oryzias latipes*	*alx3*	XM_004070679.3
Medaka	*Oryzias latipes*	*alx4a*	XM_023953432.1
Medaka	*Oryzias latipes*	*alx4b*	XM_004069555.4

Table S2. Primers used in this study.

Primer name	Sequence (5′–3′)
alx4a-gRNA-F	TAATACGACTCACTATAGCGGCCTCCGATAGGACGCCGTTTTAGAGCTAGAAATAGC
alx4b-gRNA-F	TAATACGACTCACTATACCGGGTAAAGGGCAGGCTTAGTTTTAGAGCTAGAAATAGC
gRNA-R	AGCACCGACTCGGTGCCAC
alx4a-page-F	CCAGATACCTGGCTGTGAAGG
alx4a-page-R	TTTGTTGCTTTCGCCACCAT
alx4b-page-F	ACCAAGCGAACCCTTTCAGG
alx4b-page-R	GTTGAAGGTGCCCTCCTCAG
alx4a-test-F	CATCCTTTTTGGGCAGCGTC
alx4a-test-R	TCCAGCTCTATCTGCAGTTTGTT
alx4b-test-F	CACTGTAGGCTACTGAGGCG
alx4b-test-R	TTCCCTGGAGAAATCGCACA
β-actin-qPCR-F	GGCATCACACCTTCTACAACGA
β-actin-qPCR-R	ACGCTCTGTCAGGATCTTCA
mitfa-qPCR-F	TCCATCAACAATTGTCCACCC
mitfa-qPCR-R	GGAAAGCTCTCTGGCCTTTGA
scarb1-qPCR-F	TGGTGTTGGGTGCATCAGT
scarb1-qPCR-R	ACAGGCTTGGTCAGGAAGG
tfec-qPCR-F	CTGACATGCGGTGGAACAAAG
tfec-qPCR-R	CATGTTTGGTAGCCCGTGAG
pnp4a-qPCR-F	CGCTCGCTGACACACTGAAGT
pnp4a-qPCR-R	GGGAAAGGTCGTCTTGCAGAGA
gart-qPCR-F	ACGAGATGGCGCACAAACTA
gart-qPCR-R	AGCCCGCTGACTGACTTATG
paics-qPCR-F	CGTCGCCAGAATTACAGGACT
paics-qPCR-R	GCTGGTCCAAGAGCTCGAAA
ednrba-qPCR-F	ATTGGCCATTTGGGGTGACT

Primer name	Sequence (5′–3′)
ednrba-qPCR-R	CACGGCACGATACCTGTCAA
mpv17-qPCR-F	ACGGTGAGCTGTAAAGGACG
mpv17-qPCR-R	AGACCCCGCTGTCACTATCT
ltk-qPCR-F	TAACTTCACGAGCCGGGTTT
ltk-qPCR-R	CGTGGCAGGTACTGCTCATTAT
sox10a-qPCR-F	GGCAAACTGTGGAGGCTACT
sox10a-qPCR-R	TTGCCATTTTTGCGTCTCCG
csf1ra-qPCR-F	TGGATCCACTGTTACACTCCAG
csf1ra-qPCR-R	TGCAGGCTCCAAGTAGGTTG
gch2-qPCR-F	GCAAGCTGGCAAGGATTGTG
gch2-qPCR-R	GCGCCGCTTCAATAACCAC

Note: All oligonucleotide sequences are presented in the 5′–3′ direction.

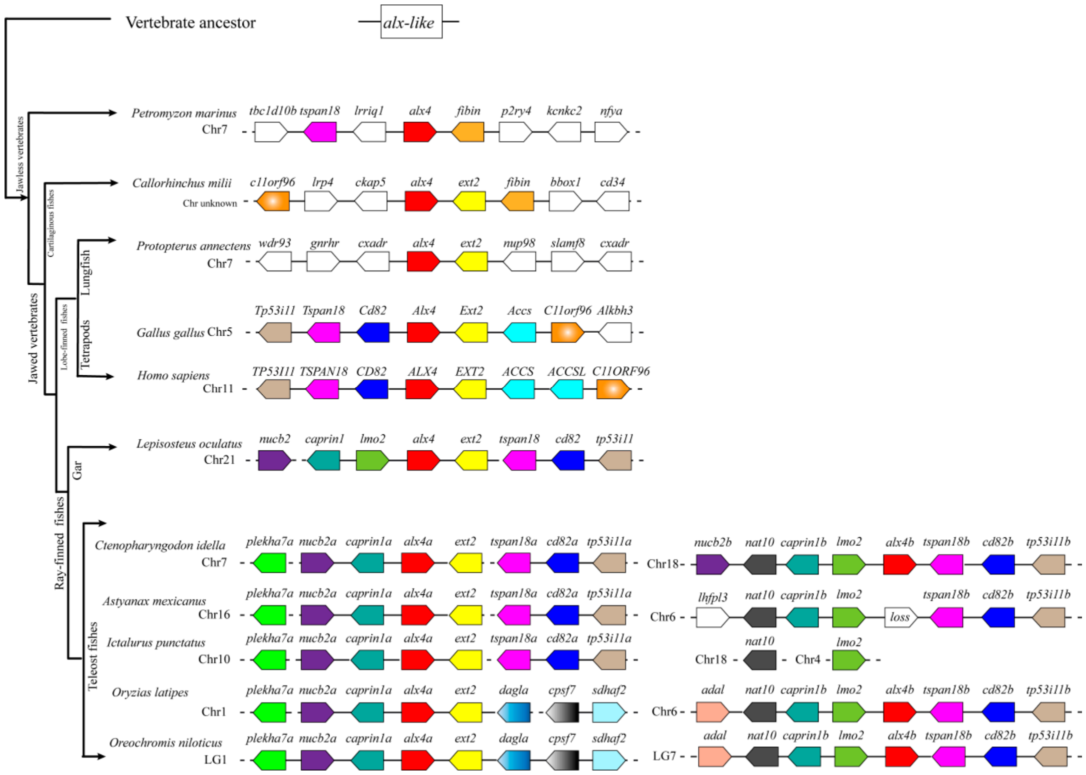

Figure S1. Chromosomal synteny of Alx4 in vertebrates. Identically colored blocks indicate homologous genes, and arrow direction indicates gene orientation. Dotted lines indicate omitted genomic regions; line lengths are not proportional to physical distance. Chr, chromosome; LG, linkage group.

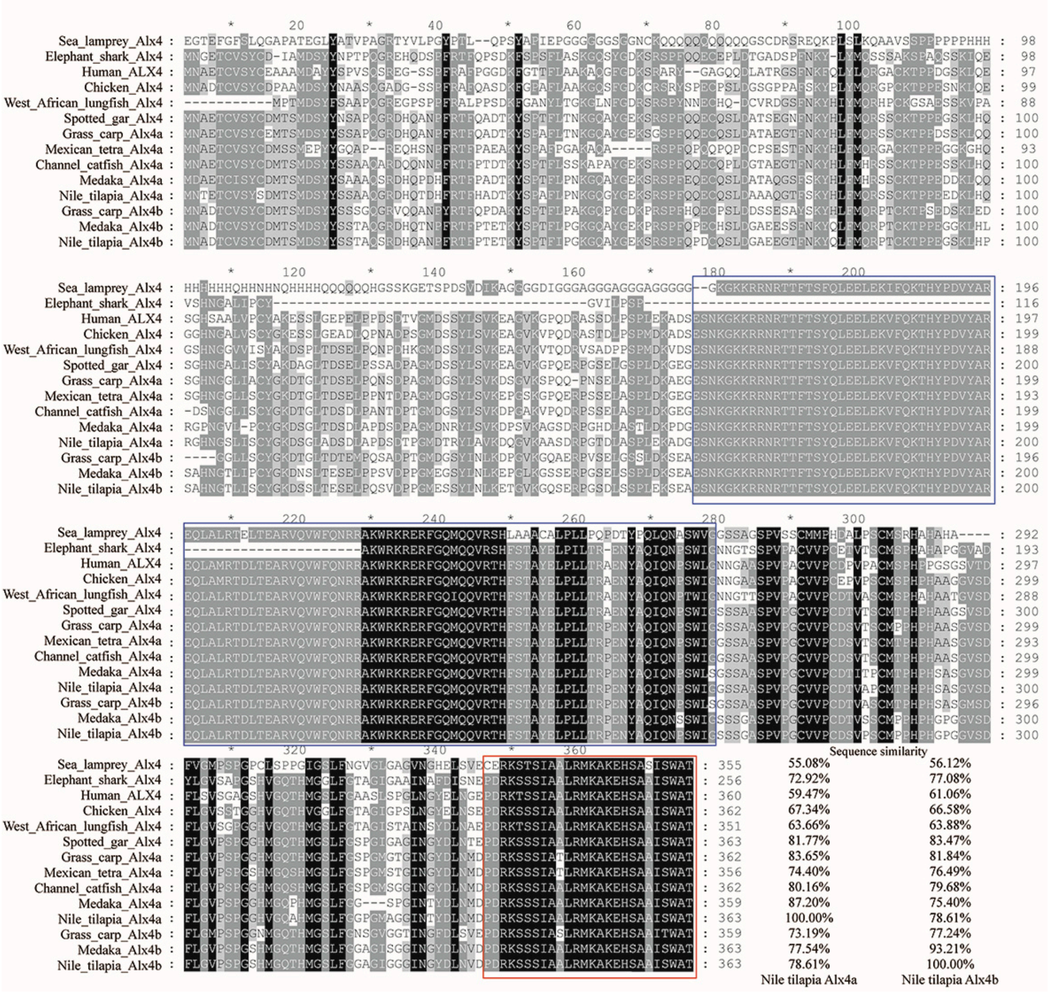

Figure S2. Comparison of vertebrate Alx4 amino acid sequences. Shading intensity indicates residue conservation. The homeodomain is outlined in blue and the OAR domain in red. Sequence-similarity values indicate the similarity of tilapia Alx4a and Alx4b to Alx4 proteins from other vertebrates.

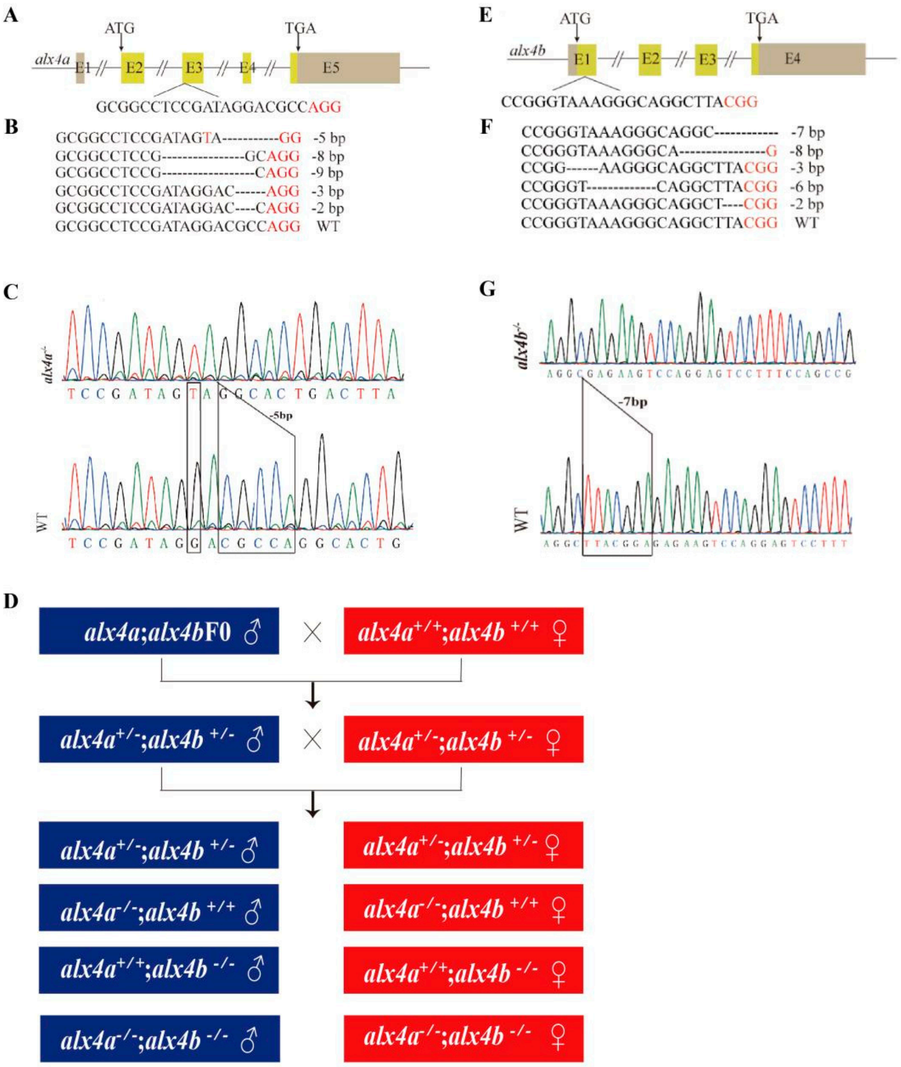

Figure S3. Establishment of the *alx4a* and *alx4b* single-mutant and double-mutant lines. (A,E) Tilapia *alx4a* and *alx4b* gene structures showing the CRISPR target and PAM sequences; yellow denotes coding regions and brown denotes untranslated regions. (B,F) Sanger sequencing of F_1 mutant alleles; PAM sequences are shown in red and dashes indicate nucleotide deletions. (C,G) Sequence validation of homozygous *alx4a* and *alx4b* mutants. (D) Breeding strategy used to establish the homozygous mutant lines.

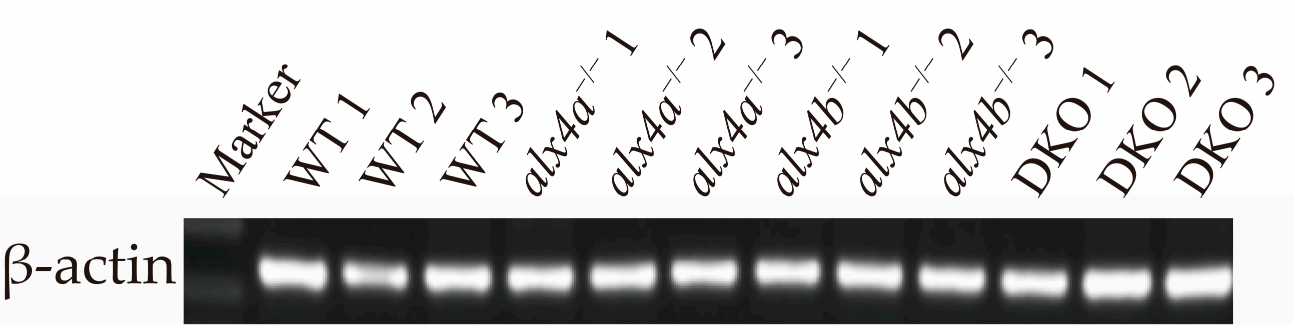

Figure S4. Evaluation of β-actin expression in dorsal skin samples used for qPCR analysis. Semi-quantitative RT-PCR analysis of β-actin was performed using the same 12 independent biological samples subsequently used for qPCR analysis, including WT, *alx4a*$^{-/-}$, *alx4b*$^{-/-}$, and *alx4a*$^{-/-}$; *alx4b*$^{-/-}$ double-knockout (DKO) fish (n = 3 independent fish per genotype). Comparable β-actin amplification was observed across the four genotypes, supporting its suitability as the internal reference gene under the experimental conditions used in this study. WT, wild type; DKO, double knockout.
